# Simultaneous Nasal Carriage by Methicillin-Resistant and Methicillin Susceptible *Staphylococcus aureus* of Lineage ST398 in a Live Pig Transporter

**DOI:** 10.3390/pathogens9050401

**Published:** 2020-05-21

**Authors:** Paula Gómez, Carmen Aspiroz, Nazreen F. Hadjirin, Daniel Benito, Myriam Zarazaga, Carmen Torres, Mark A. Holmes

**Affiliations:** 1 Area of Biochemistry and Molecular Biology, University of La Rioja, 26006 Logroño, Spain; paula_gv83@hotmail.com (P.G.); danielbenitopascual@gmail.com (D.B.); myriam.zarazaga@unirioja.es (M.Z.); 2 Department of Microbiology, Hospital Royo Villanova, 50015 Zaragoza, Spain; caspirozs@gmail.com; 3 Department of Veterinary Medicine, University of Cambridge, Cambridge CB3 0ES, UK; nh396@cam.ac.uk (N.F.H.); mah1@cam.ac.uk (M.A.H.)

**Keywords:** MRSA, SCC*mec* remnant, ST398, whole genome sequence, pig worker

## Abstract

Methicillin-resistant *Staphylococcus aureus* (MRSA) sequence type (ST)398 is a livestock associated (LA) lineage with zoonotic potential, especially in humans with live pig contact. The objective of this study was to characterize two *S. aureus* strains of lineage ST398 (one methicillin-resistant (MRSA), one methicillin-susceptible (MSSA)) isolated from the same nasal sample of a patient admitted in the Intensive-Care Unit of a Spanish Hospital, and with previous occupational exposure to live pigs, by whole-genome-sequencing (WGS). The sample was obtained during routine surveillance for MRSA colonization. Purified genomic DNA was sequenced using Illumina HiSeq 2000 and processed using conventional bioinformatics software. The two isolates recovered were both *S. aureus* t011/ST398 and showed similar resistance-phenotypes, other than methicillin susceptibility. The possession of antibiotic resistance genes was the same, except for the *mec*A-gene located in SCC*mec*V in the MRSA isolate. The MSSA isolate harbored remnants of a SCC*mec* following the deletion of 17342bp from a recombination between two putative primases. Both isolates belonged to the livestock-associated clade as defined by three canonical single-nucleotide-polymorphisms, and neither possessed the human immune evasion cluster genes, *chp*, *scn*, or *sak.* The core genome alignment showed a similarity of 99.6%, and both isolates harbored the same mobile genetic elements. The two nasal ST398 isolates recovered from the patient with previous occupational exposure to pigs appeared to have a livestock origin and could represent different evolutionary steps of animal-human interface lineage. The MSSA strain was formed as a result of the loss of the *mec*A gene from the livestock-associated-MRSA lineage.

## 1. Introduction

Methicillin-resistant *Staphylococcus aureus* (MRSA) belonging to the sequence type (ST)398 is a livestock-associated (LA) lineage [1] found in people and animals, demonstrating little host species specificity. People having contact with farm animals or living close to a farm are at higher risk for carriage of this opportunistic pathogen. Phylogenetic studies showed that this MRSA ST398 probably originated as a methicillin-susceptible *S. aureus* (MSSA) lineage found in humans, which subsequently spread to animals, where it acquired methicillin resistance [2]. Human and animal ST398 strains belonging to human and livestock clades, respectively, can be discriminated using three canonical single-nucleotide polymorphisms (SNPs) [3]. The carriage of an animal lineage ST398 MRSA by a human subject is likely to have resulted from zoonotic transmission, either directly or indirectly. This lineage now seems to ready colonize both people and livestock, and represents a significant proportion of human MRSA isolates in regions with a high density of pig farms.

During routine hospital surveillance to detect *S. aureus* and MRSA colonization in the Intensive-Care Unit (ICU) of a Spanish Hospital, a nasal sample of a noninfectious disease patient, who worked as a truck driver transporting live pigs, was cultured. Two *S. aureus* ST398 strains were detected with identical pulsed-field gel electrophoresis profiles and similar phenotypic characteristics, other than one being methicillin-susceptible and one being methicillin-resistant. The aim of this study was to investigate the phylogeny and the genetic differences between these two isolates.

## 2. Results and Discussion 

### 2.1. Identification of the Isolates, Molecular Typing, and Antibiotic Resistance Phenotype

The two isolates recovered from the nasal sample of the pig truck driver were confirmed as *S. aureus*, one MRSA (strain C6828) and the other MSSA (strain C6829). Both strains were typed as ST398/*spa*-type t011/ and were tetracycline-, clindamycin-, gentamicin-, tobramycin-, and kanamycin-resistant.

### 2.2. Whole Genome Sequencing Results 

The sequence data from C6828 and C6829 assembled to 68 and 52 contigs from a yield of 2.85 GBp and 2.83 GBp, respectively, had a coverage of approximately 100-fold. Multilocus sequence typing of the resulting assembly confirmed them to be ST398 and was used to identify the livestock-associated lineage canonical SNPs. The genome data were placed in the European nucleotide archive (http://www.ebi.ac.uk/ena) under accession numbers ERS659527 and ERS659528.

### 2.3. Virulence and Antimicrobial Resistance Genotype

The two strains carried the following antimicrobial resistance genes: *blaZ*, *tet*(M), *norA*, *lnu*(B), *dfrK*, *aadE*, *aac*(6′)-*aph*(2′′), and *aadD*. The gene *mecA* was only detected in MRSA strain C6828, and *tet*(L) was found in the two strains. In strain C6828, only 902 of 1377 nucleotides (66%) were present. It is important to note that the *tet*(M) gene is strongly linked with livestock-associated ST398 lineage, and that neither strain possessed genes from the immune evasion cluster such as the *scn, chp*, or *sak* genes, which are considered as human host genetic markers [2,3]. Resistance against lincosamides, aminoglycosides, and trimethoprim seems to be a frequent finding in the livestock lineage, and fluoroquinolone-resistance has also been described [1]. Lastly, according to the three canonical SNPs described by Stegger et al., these strains belonged to the livestock clade. The results indicate that, despite the fact that both strains were recovered from the same patient, they seem to be from a livestock origin, but C6829 has lost some characteristics, notably the presence of *mecA* gene.

The detection of virulence genes showed that both strains harbored the same content: *hlb* (β-hemolysin), *hlgAB* and *hlgCB* (γ-hemolysins), and *aur* (aureolysin), which were encoded in the core genome of *S. aureus*.

### 2.4. Comparison between Both Strains

The pan-genome study showed a 99.6% similarity in core genome alignment between the strains and 2615 genes were common to both isolates. It has been reported that the core genome is largely preserved within the same lineage [4]. Otherwise, 20 insertions-deletions (indels, all in frame) and 134 SNPs from CDS (43 of them nonsynonymous) were also detected in the alignment (see Supplementary Table S1). Differences in *clfB* and *sdrD* were also found. The protein product of *clfB* gene (ClfB) is an adhesion factor which plays an important role in the colonization of nasal epithelium and has been proposed as a marker of microvariation [5]. The protein product of *sdrD* gene (serine-aspartate repeat-containing protein D), as well as ClfB, are involved in the adhesion to the extracellular matrix of the host [6]. In our strains, MSSA C6829 presented 24 additional Ser-Asp repetitions compared to MRSA C6828. 

**Figure 1:**
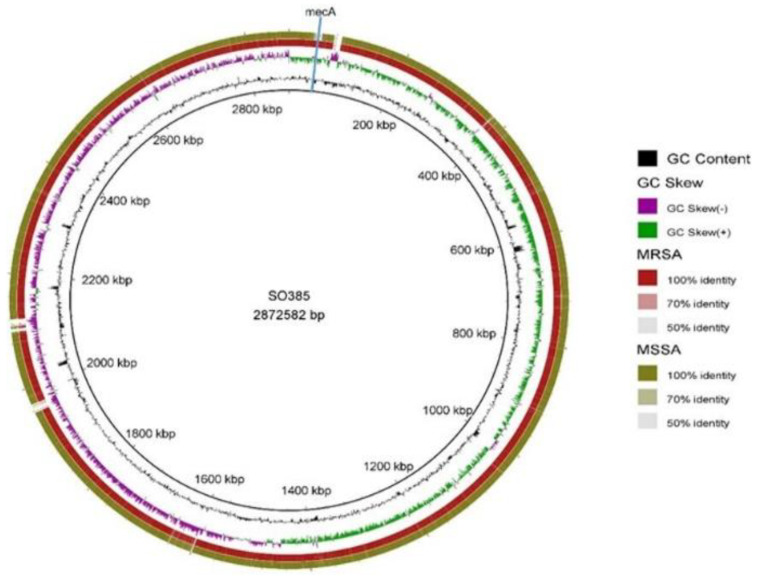
Circular comparison of C6828 (MRSA) and C6829 (MSSA) genomes using SO385 as reference. The green ring represents the C6829 strain and the red ring C6828. The position of *mecA* gene in C6828 is indicated by blue line. % GC content and GC Skew are represented in innermost circles (colors indicated in the coded legend).

On the other hand, 40 genes were unique to one or the other isolate, 11 in C6829, and 29 in C6828 (see Supplementary Table S2). The MSSA strain harbored a *sdrE* gene that was not present in the MRSA strain, the product of which binds to the complement regulator factor H, avoiding the host defenses [7]. Despite the fact that no single gene has been described as essential for the colonization of humans or pigs, the distribution of mobile genetic elements (MGE) is variable and may be important in adaptation to different environments [4]. Figure 1 shows the comparison between both strains using the reference genome SO385, revealing that the strains were nearly identical, except for the differences associated with the *mecA* and adjacent genes (present only in C6828), which was included in the MGE staphylococcal cassette chromosome *mec* (SCC*mec*). The pan-genome study revealed that 18 of the 40 genes, which were present in one strain and not in the other (and vice versa), belonged to SCC*mec* (see Supplementary Table S2).

**Figure 2:**
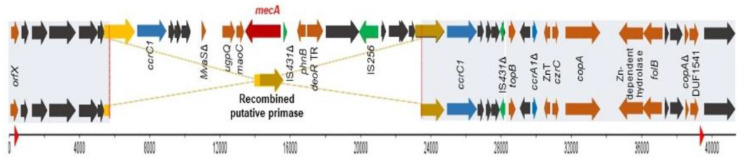
Schematic representation of the comparison between SCC*mec*V (C6828, MRSA) in the top and the SCC*mec* remnant (C6829, MSSA) in the bottom. Red arrow indicates *mecA* gene, *ccr* genes are in blue, hypothetical proteins in grey, and insertion sequences (IS) are indicated in green. The red triangle in the bp scale represent the position of direct repeats. The grey boxes show the coverage between both strains. The scheme represents the recombination between the two putative primases, giving as a result one deletion in C6829.

Both strains aligned partially with SCC*mec*V (5C2&5)c and differed only in a gap of 17342bp, present in the C6829 strain, because of a putative primase recombination (Figure 2). The presence of SCC*mec* remnants has been previously described in *S. aureus* CC398 isolates of humans and animals [8,9], as well as in human isolates of other lineages [10,11], suggesting potential evolutionary and adaptive steps. Moreover, the *mecA* gene can be lost during the storage process [12]. The conversion from MRSA to MSSA has been reported as a result of a recombination event between *ccrC* genes [8,9]. In addition, it has been described that *IS431* elements could be involved in the deletion of SCC*mec* fragments in MRSA isolates, in parallel to the acquisition of a vancomycin-intermediate-resistance phenotype [10,13]. 

In regard to the other MGEs, the same content was found in both strains. Examination of the phage content resulted in the identification of two intact and two defective prophages in each strain. One of these intact prophages showed similarity with StauST398-3, and the other with *phi*SA2. The plasmid *rep* genes *rep22* and *rep21* were present and associated with *rep*B (pUB110) and pSO385-3, respectively. Two potential pathogenic islands, νSaα and νSaγ, were found. The phylogenetic context of the two strains among previously sequenced ST398 isolates is presented in Supplementary Figure S1, which shows that the isolates are very closely related to the other European isolates in the livestock lineage clade and both strains cluster together. 

It is also of interest that, while the *mecA* gene is known to result in a fitness cost [14], both strains appeared to coexist within the same human host without the MSSA version outcompeting its MRSA counterpart.

## 3. Materials and Methods 

### 3.1. Sample Collection, Isolation and Identification

A nasal swab was taken from a male patient who was admitted at the ICU of a Spanish hospital as a result of cardiac disease with no evidence of infectious disease in 2014. This patient had regular contact with livestock through his work as the driver of a truck transporting pigs. The sample was inoculated onto blood agar (5% sheep blood), colistin nalidixic agar, and Brillance MRSA agar (Oxoid, Basingstoke, UK) plates, and then incubated at 35 °C for 24 h. During the screening process, it was noticed that colonies with two distinctly different *S. aureus* morphologies grew on both blood agar and colistin nalidixic agar plates. These differences were related to the expression of beta-hemolysis. Representative colonies with these two morphologies were recovered from blood agar plates (detected approximately in a proportion 1:1), and the species confirmed by amplification of the species-specific *nuc* gene [15]. 

### 3.2. Molecular Typing and Antimicrobial Susceptibility Testing

The two recovered *S. aureus* isolates were characterized by *spa*-typing (www.ridom.com) and by multilocus-sequence-typing (MLST) to determine the sequence type (ST) (www.mlst.net). Pulsed-field gel electrophoresis was performed according to a previously described protocol [16].

Antimicrobial susceptibility to penicillin, cefoxitin, tetracycline, erythromycin, clindamycin, gentamicin, kanamycin, tobramycin, streptomycin, trimethoprim/sulfamethoxazole, mupirocin, and fusidic acid was performed using the disk-diffusion method (Version 5.0., http://www.eucast.org).

### 3.3. Whole Genome Sequencing and Analysis of Sequences

Purified whole genomic DNA was obtained using the MasterPure^TM^ DNA Purification Gram Positive kit (Cambio, Cambridge UK), and Illumina Library preparation was performed according to methods previously described [17]. The genomes were sequenced on an Illumina HiSeq 2000 platform at the Welcome Trust Sanger Institute, Cambridge, UK, using 125-bp paired-end sequencing. *De novo* assembly and initial annotation was carried out using the bioinformatic pipelines available at the Welcome Trust Sanger Institute [18]. The reordering of the contigs was performed by alignment against the SO385 genome (GenBank accession number: AM990992) using Mauve v2.4.0 [19].

Predicted coding sequences (CDS) were identified and annotated automatically using RAST (Rapid Annotations using Subsystems Technology) [20] and manually with Artemis [21]. The common and unique genes of the two genomes were identified using Roary v3.7.1 [22]. BLAST-Ring-Image-Generator (BRIG) [23] was employed to compare the two genomes visually (using SO385 as the reference genome). The resistance and virulence genotypes, as well as the presence of *rep* genes, were identified using ResFinder v2.1 [24], VirulenceFinder v1.5 [25], and PlasmidFinder v1.3 [26], respectively. Local BLAST was performed to find Pathogenicity Islands according with the Pathogenicity Island Database (PAIDB, http://www.paidb.re.kr). PHAge Search Tool was run to determine the presence of prophage sequences [27].

### 3.4. Phylogenetic Analysis

In order to understand how strains C6828 and C6829 clustered among a collection of 89 previously sequenced ST398 *S. aureus* strains, a SNP-based maximum likelihood phylogenetic tree was reconstructed using RAxML v8.2.10 [28]. Briefly, FastQ files from the study isolates, the 89 previously published ST398 isolates, and MRSA252 (used here as the outgroup) were mapped against the SO385 reference genome using SMALT (http://www.sanger.ac.uk/resources/software/smalt/) to identify SNPs, which were then used to generate the phylogenetic tree. RAxML implemented a GTR Gamma substitution model and rapid bootstrapping.

## 4. Conclusions

The comparison of the two *S. aureus* ST398-t011 strains recovered from a patient with professional contact with pigs, demonstrates a spontaneous genomic event in the evolution of this wild-type LA-ST398 isolate. It provides valuable information helping our understanding the evolution of this microorganism that exists in the animal-human interface. 

## Supplementary Materials

The following are available online at https://www.mdpi.com/2076-0817/9/5/401/s1, Figure S1: Phylogenetic tree showing the two strains sequenced in this study (C6828 and C6829, highlighted with red quadrangle) in the context of previously sequenced ST398 isolates. Table S1: Single nucleotide polymorphisms (SNPs) and insertions or deletions (INDELs) in the core genome alignment of C6828 (MRSA) and C6829 (MSSA) isolates. Table S2: Unique genes in MRSA (C6828) and unique genes in MSSA (C6829) isolates with coverage and identity compared to the same features in the SO385 genome indicated.
